# Effects of lifestyle and its interaction with anemia on cognitive function in older adults: A longitudinal study

**DOI:** 10.1002/pchj.712

**Published:** 2023-12-17

**Authors:** Jia Yang, Chen Zhou, Hui‐Jie Li

**Affiliations:** ^1^ CAS Key Laboratory of Behavioral Science Institute of Psychology Beijing China; ^2^ Department of Psychology University of Chinese Academy of Sciences Beijing China; ^3^ Zhejiang Museum of Natural History Hangzhou China

**Keywords:** anemia, cognitive function, lifestyle factor, longitudinal change

## Abstract

A better understanding of the impact of lifestyle factors on cognitive function in older adults is critical for developing intervention strategies to achieve successful aging. Moreover, older adults who fulfill the World Health Organization criteria for anemia have a significantly higher risk of developing dementia. In the current study, we aimed to assess the buffering effects of lifestyle on cognitive function in older Chinese adults through a nationally representative survey. The sample consisted of 1201 participants (mean age: 82.39 ± 12.08 years, 52.1% female) from the 2011/2012 and 2014 waves of the Chinese Longitudinal Healthy Longevity Survey. Multiple linear regression analyses were used to explore the relationship between changes in lifestyle factors and the rate of cognitive function changes, as well as the effects of the interaction between lifestyle factors and anemia on cognitive function changes. Increased levels of participation in leisure activities, social activities, and dietary diversity delayed cognitive decline. Persistent anemia accelerated cognitive decline, while frequent participation in leisure activities delayed cognitive decline due to anemia. The increased levels of participation in leisure activities, social activities, and dietary diversity can alleviate the cognitive decline caused by aging itself, and more frequently participation in leisure activities can also alleviate the adverse effects of anemia on cognitive function in older adults.

## INTRODUCTION

By the end of 2020, China's population aged 65 or over had reached 190 million (National Bureau of Statistics, 2021). Cognitive decline is prevalent in older adults, and faster cognitive decline leads to higher rates of dementia (Lipnicki et al., 2017). Because cognitive decline is an inevitable part of aging, it is crucial to explore effective approaches to slow cognitive decline (Livingston et al., 2017).

Cognitive health is critical for older adults to achieve healthy aging. Previous studies have summarized a variety of factors associated with cognitive health, including sex, genetics, occupation, early life experiences, socioeconomic status, brain trauma, disability, and lifestyle (Lee et al., 2020; Marioni et al., 2014). Among them, lifestyle has received increasing attention because of its directly modifiable characteristics. While there is no widely accepted definition of lifestyle, researchers generally believe that lifestyle includes alcohol and tobacco use, diet, physical activities, leisure activities, and social participation (Freudenberg, 2012). Lifestyle changes, including increased leisure activities, social activities, physical exercise and dietary diversity, and reduced smoking or alcohol consumption, can reduce or delay the risk of cognitive impairment (Clare et al., 2017; Liu et al., 2020). Older adults who are heavily involved in social and leisure activities tend to live in environments with rich stimulation and cognitive demands, which can prevent cognitive decline and improve quality of life (Karp et al., 2006; Mao et al., 2020; Marioni et al., 2015). As older adults age, the positive relationship between activity participation and cognitive function becomes stronger (Hultsch et al., 1993). Existing evidence suggests that middle‐aged and older adults with higher engagement in physical exercise have fewer age‐related alterations in Aβ deposition, glucose metabolism of the precuneus, and hippocampal gray matter volume (Liang et al., 2010; Okonkwo et al., 2014). In addition, older adults who are engaged in a variety of leisure activities have greater hippocampal gray matter volume (Iizuka et al., 2021).

As an important part of lifestyle, dietary diversity is associated with lower risks of cognitive decline and dementia, possibly because individuals with higher dietary diversity have fewer vascular risk factors and lower oxidative stress (Zheng et al., 2021). In contrast, smoking and excessive alcohol consumption may increase oxidative stress and induce cardiovascular disease, thereby increasing the risk of dementia (Livingston et al., 2017). Encouraging older adults to engage in leisure, social, and physical activities, as well as to have a healthy diet, can enhance cognitive reserve and help maintain cognitive health, while smoking has no beneficial effect on cognitive function (Clare et al., 2017).

Although previous studies have explored the relationship between lifestyle and cognitive function, many of them explored only the effects of certain lifestyle factors on cognitive function rather than taking a more comprehensive view of lifestyle. More importantly, few studies have revealed the dynamic relationship between lifestyle changes and cognitive function changes in older adults. A healthy lifestyle can have a significant impact on healthy aging and longevity, even in later life (Dreher, 2018). Predicting changes in cognitive function through dynamic lifestyle changes can more directly reflect the special protective effects of lifestyle in older adults.

Chronic diseases associated with aging, such as anemia, are also considered risk factors for cognitive decline (Tan et al., 2018). Anemia is a common clinical symptom of a decreased volume of human peripheral red blood cells below the lower limit of the normal range. It is a global public health problem affecting all ages (Cappellini & Motta, 2015). The prevalence of anemia increases from 16.1% to 57.1% in adults aged 40 to 100 years (Zhai et al., 2010). Older adults with anemia have lower episodic memory, working memory, and slower processing than those without anemia (Jonassaint et al., 2014; Qin et al., 2019). Longitudinal studies have also shown that older adults who fulfilled the World Health Organization (WHO) criteria for anemia had a significantly higher risk of developing dementia (Hong et al., 2013). Lifestyle factors, including malnutrition, smoking, alcohol consumption, and insufficient exercise, are considered important risk factors for anemia, which increases the risk of cognitive impairment (Paramastri et al., 2021). A recent study revealed that older Japanese women with lower scores on the Montreal Cognitive Assessment experienced a higher risk for anemia 3 years later, and researchers suggested that a healthy lifestyle might be important to prevent anemia (Noma et al., 2023). However, whether a healthy lifestyle in older adults can alleviate the adverse effects of anemia on cognitive function has not yet been investigated.

In this longitudinal study, by using a nationally representative survey, we aimed to address the dynamic relationship between comprehensive lifestyle changes and changes in cognitive function and the moderating effect of lifestyle changes on the relationship between anemia and changes in cognitive function. Cognition refers to the mental processes involved in acquiring knowledge and understanding (VandenBos, 2007), and the classification of cognition varies widely among different researchers. In the field of cognitive aging, many researchers have used the Mini‐Mental State Examination (MMSE) score as an indicator of global cognition function (Bai et al., 2023; Gao et al., 2017; Yu et al., 2021). According to the environmental complexity hypothesis, a socially intensive lifestyle may provide cognitive benefits to older adults (Greenough et al., 1986; Schooler & Mulatu, 2001). Therefore, we first hypothesized that a healthy lifestyle could slow the process of cognitive decline. Moreover, we hypothesized that a healthy lifestyle could buffer the adverse effects of anemia on cognitive function in older adults.

## MATERIALS AND METHODS

### Data source and participants

**FIGURE 1:**
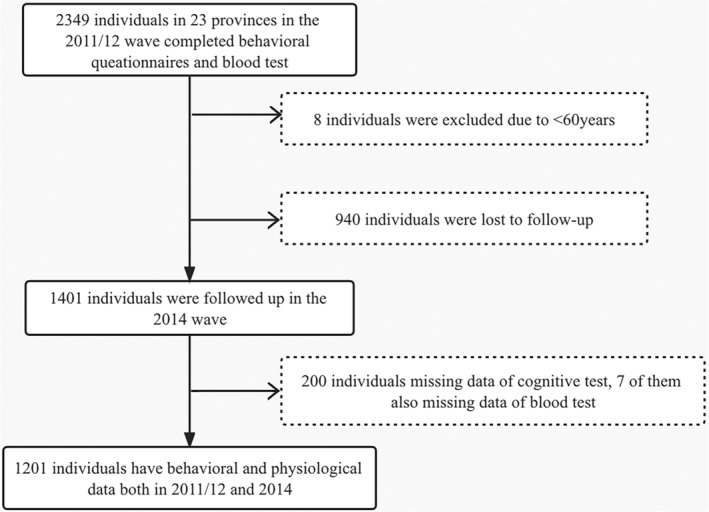
Flowchart of the study sample.

The data used in the current study are from the 2011/12 and 2014 waves of the Chinese Longitudinal Healthy Longevity Survey (CLHLS), which is an ongoing, prospective cohort study of community‐dwelling Chinese older adults. The CLHLS began in 1998, and surveys are carried out every 2–3 years. The CLHLS obtained ethics approval from the Research Ethics Committee of Peking University (IRB00001052‐13074) and received written informed consent from all participants. Briefly, 2349 participants aged 60 years and over completed behavior questionnaires and blood tests in the 2011/12 wave. Of those, 1401 participants were followed up during the 2014 wave. Finally, 1201 participants completed the behavior questionnaires and blood tests in two waves. Details about sample selection are presented in Figure 1.

### Measures

#### Cognitive function

Global cognition was measured using the Chinese Mini‐Mental State Examination (CMMSE). Previous studies have validated the CMMSE, and Cronbach's α values were .94 (Zhang, 2006) and .98 (Zhang et al., 2010). Twenty‐four items were included in the CMMSE, and these items focus on seven cognitive domains: Orientation, Registration, Naming Foods, Attention and Calculation, Copying a Figure, Recall, and Language. The total CMMSE score ranges from 0 to 30, and a higher CMMSE score indicates better cognitive function. Cognitive decline was assessed using the rate of change in the CMMSE score, which was calculated by subtracting the baseline CMMSE score from the follow‐up CMMSE score and dividing it by the number of years of follow‐up (Lv et al., 2019). A score below zero indicates cognitive decline, a score equal to 0 indicates no cognitive change, and a score above zero indicates cognitive improvement.

#### Lifestyle factors

We aimed to analyze the lifestyle factors included in the CLHLS, including social activities, leisure activities, exercise, dietary diversity, smoking, and alcohol consumption. The factors were defined and classified according to previous studies using CLHLS data (Yin et al., 2012, 2017).

#### Social activities

Social activities include engaging in housekeeping and participating in organized social activities. Two questions were considered to assess social activities: *“Do you do housekeeping? (i.e., cooking, babysitting)*” and “*Do you attend organized social activities?*” The response was measured on a five‐point scale: 5‐almost every day, 4‐at least once per week, 3‐at least once per month, 2‐occasionally, and 1‐rarely or never. Social activity scores range from 2 to 10, with higher scores indicating higher levels of participation (Wang et al., 2021; Yin et al., 2017).

#### Leisure activities

Leisure activities include growing vegetables, watching TV or listening to the radio, working in the garden, keeping animals/pets, reading books/newspapers, and playing cards or mahjong. The questions were “*Do you grow vegetables or do other field work at present*?”, “*Do you watch TV or listen to the radio at present*?”, “*Do you do garden work*?”, “*Do you keep domestic animals/pets at present*?”, “*Are you currently participating in or engaging in reading books/newspapers*?” and “*Are you currently participating in or engaging in playing cards or mahjong*?”. The responses to these six questions were also measured on a five‐point scale. Leisure activity scores range from 6 to 30, with higher scores indicating higher levels of participation (Wang et al., 2021; Yin et al., 2017).

#### Exercise

Physical exercise was assessed with the following question: “*Do you currently participate in physical exercise? (e.g., walking, playing football, basketball or volleyball, and running)*” The responses were dichotomized as “yes” or “no” (Gu & Zeng, 2004).

#### Dietary diversity

The frequency of intake was collected for various food categories, including vegetables, fruits, legumes and their products, nuts, meat, eggs, fish, milk and dairy products, and tea. The frequency of food intake in each category was measured on a five‐point scale: almost every day, at least once per week, at least once per month, occasionally, and rarely or never. If the answer to a category was “almost every day” or “at least once per week”, a point was given; otherwise, it was zero (Yin et al., 2017). The dietary diversity scores range between 0 and 9, and a higher score indicates better dietary diversity.

#### Smoking and alcohol consumption

According to the answers to the question “*Are you smoking now?*”, participants were classified as “nonsmokers” (never or not current smokers) or “current smokers”. Drinking was assessed with a similar question, and participants were classified as “nondrinkers” (never or not current drinkers) or “current drinkers” (Mao et al., 2020).

#### Blood test

In total, 7 mL (5 + 2 mL) of blood samples were collected from all participants in heparin anticoagulation blood collection tubes. Blood samples were centrifuged within 1 hour after blood collection to isolate plasma from blood cells. Heparin anticoagulant blood samples were centrifuged at 3000 rpm for 10 min at 18°–25°C. Then, all plasma, white blood cells, whole blood, and urine samples were stored in a freezer at −80°C in the district Centers for Disease Control. The samples were then transported to the Central Clinical Laboratory of Capital Medical University in Beijing for further analyses in a 20°C transport box provided by the Chinese Centers for Disease Control (Qin et al., 2019). According to the WHO criteria, anemia is defined as a hemoglobin concentration of less than 12.0 g/dL in women and of less than 13.0 g/dL in men (WHO, 2011).

#### Confounding variables

Socioeconomic status, health status, and biomarkers associated with cognitive function were used as confounding variables (Jia et al., 2021; Lee et al., 2015; Qin et al., 2019). The socioeconomic status variables included age, sex, marital status (married, nonmarried), economic status (poor, others), and year of education (≤6 years, > 6 years) (Lee et al., 2015). Health status was composed of self‐reported health (a five‐point scale, with a higher score indicating good health status), abdominal obesity, activities of daily living (ADL), and instrumental activities of daily living (IADL). Abdominal obesity was defined based on waist circumference: greater than 90 cm for men and 85 cm for women (Qin et al., 2019). ADL includes six items that are needed to live a basic life (e.g., bathing, eating), and IADL includes eight items that are needed to live an independent life (e.g., shopping, cooking). ADL and IADL were measured on a 3‐point scoring system: do not have difficulty, need help, cannot do it. Thus, ADL scores range from 6 (least difficulty and best physical health) to 18 (most difficulty and worst physical health), and IADL scores range from 8 to 24 (Chen et al., 2020). Biomarkers included total cholesterol (TC), triglyceride (TG), and superoxide dismutase (SOD).

#### Statistical analyses

We examined the differences of participants in variables at baseline (2011/2012 wave) and follow‐up (2014 wave) with paired *t*‐tests and McNemar's chi‐square test. Changes were assessed using the rate of change in leisure activities, social activities, and dietary diversity, which was calculated by subtracting the baseline score from the follow‐up score and dividing it by the number of years of follow‐up. A score below zero indicates a decline, a score equal to 0 indicates no change, and a score above zero indicates improvement. Given that smoking, drinking, and exercise are dichotomous scoring forms (yes or no), we divided the changes in these three activities into four groups based on changes from baseline (T1) to follow‐up (T2): Yes_T1_‐Yes_T2_, Yes_T1_‐No_T2_, No_T1_‐No_T2_, and No_T1_‐Yes_T2_. Subsequently, we used multiple linear regression to test the prediction of the cognitive changes by lifestyle factors. Two sets of regression models were used: Model 1 was the basic model without any confounding variables, while Model 2 was adjusted for all potential confounders. We further used multiple linear regression to examine the effect of the interactions between lifestyle factors and anemia on cognitive changes and used the above two sets of regression models. All statistical analyses were performed using SPSS statistical software package version 22.0. All *p* values were two‐sided.

## RESULTS

### Descriptive statistics

Table 1 shows the characteristics and differences of participants in variables at baseline and follow‐up. At baseline, the participants were 82.39 ± 12.08 years old, and 52.1% were women (Table 1). Participants reported relatively high levels of participation in leisure activities (13.47 ± 5.14), social activities (4.77 ± 1.94), and good dietary diversity (4.26 ± 1.97) at baseline, while the proportions of exercise (15.5%), current smokers (19.5%) and drinkers (16.9%) were lower. The prevalence of anemia was 44.4% at baseline (Table 1).

**TABLE 1:** Characteristics of participants at baseline and follow‐up.

Variables	Baseline	Follow‐up	*p*
Age	82.39 ± 12.08	84.44 ± 12.05	<.001
Sex (female)	626 (52.1%)	626 (52.1%)	
Education (≤6)	1070 (89.6%)	1067 (89.4%)	.375
Marriage (Married)	563 (47.1%)	522 (44.3%)	<.001
Economics (poor)	121 (10.2%)	109 (9.2%)	.419
MMSE score	25.19 ± 7.33	23.66 ± 8.42	<.001
Dietary diversity	4.26 ± 1.97	4.41 ± 1.94	<.05
Leisure activities	13.47 ± 5.14	13.19 ± 5.58	.061
Social activities	4.77 ± 1.94	4.49 ± 2.07	<.001
Smoking (Current smoker)	231 (19.5%)	191 (16.0%)	<.001
Alcohol drinking (Current drinker)	201 (16.9%)	179 (15.1%)	.156
Exercise	181 (15.5%)	181 (15.4%)	.801
Health status			
Self‐report health	3.41 ± 0.77	3.42 ± 0.79	.876
ADL	6.38 ± 1.38	6.78 ± 2.21	<.001
IADL	11.40 ± 4.95	12.61 ± 5.86	<.001
Abdominal obesity (Yes)	277 (23.3%)	273 (23.1%)	.608
Biomarkers			
TC	4.35 ± 0.95	4.78 ± 1.02	<.001
SOD	57.20 ± 9.85	57.05 ± 8.67	.656
Hgb	12.73 ± 2.26	12.81 ± 1.97	.066
Anemia (Yes)	519 (44.4%)	455 (38.1%)	<.001
TG	1.03 ± 0.67	1.27 ± 0.80	<.001

*Note*: Continuous variables, mean ± SD; categorical variables, *n* (%).

Abbreviations: ADL, activities of daily living; Hgb, hemoglobin concentration; IADL, instrumental activities of daily living; SOD, superoxide dismutase; TC, total cholesterol; TG, triglyceride.

The average cognitive function score, measured by CMMSE, was 25.19 points at baseline but decreased to 23.66 points at follow‐up (Table 1). Compared with baseline, the level of participation in social activities decreased significantly at follow‐up (*p* < .001), and the proportions of current smokers and anemia also decreased significantly (*p* < .001). During the follow‐up, the dietary diversity increased (*p* < .05), while there were no significant changes in the level of participation in leisure activities or the proportions of exercise or alcohol drinking (Table 1).

### Association between lifestyle changes and cognitive function change rate

Considering that cognitive decline was assessed using the rate of change in CMMSE scores, we used multiple linear regression analyses to investigate the relationship between changes in lifestyle factors and the rate of change in CMMSE scores. The unadjusted model (Model 1) showed that increased participation in leisure activities (*β* = 0.077, 95% CI [95% confidence interval]: [0.015, 0.189], *p <* .05), social activities (*β* = 0.101, 95% CI: [0.120, 0.542], *p <* .01), and dietary diversity (*β* = 0.082, 95% CI: [0.057, 0.427], *p <* .05) delayed cognitive decline (Table 2). Compared with participants who never exercised, participants who began to exercise in their later life could also delay cognitive decline (*β* = 0.070, 95% CI: [0.072, 1.443], *p <* .05). Moreover, after adjusting for all confounding variables (Model 2), multiple linear regression analyses found that increased participation in leisure activities (*β* = 0.065, 95% CI: [0.001, 0.171], *p <* .05), social activities (*β* = 0.091, 95% CI: [0.087, 0.510], *p <* .01), and dietary diversity (*β* = 0.077, 95% CI: [0.045, 0.409], *p <* .05) still delayed cognitive decline (Table 2). After adjusting for confounding variables, the effect of exercise became insignificant (Table 2). Smoking and alcohol consumption changes were not significantly associated with cognitive function changes with or without controlling for confounding variables (Table 2). The results support the first hypothesis that a healthy lifestyle (increased participation in leisure activities, social activities, and dietary diversity) can delay cognitive decline in older adults.

**TABLE 2:** Multiple linear regressions between lifestyle factor changes and cognitive changes in older adults.

Variable	Model 1			Model 2		
	*β*	95% CI	*p*	*β*	95% CI	*p*
Leisure activities	0.077	0.015, 0.189	<.05	0.065	0.001, 0.171	<.05
Social activities	0.101	0.120, 0.542	<.01	0.091	0.087, 0.510	<.01
Dietary diversity	0.082	0.057, 0.427	<.05	0.077	0.045, 0.409	<.05
Smoking						
No_T1_‐No_T2_		Reference			Reference	
No_T1_‐Yes_T2_	0.002	−1.399, 1.474	.959	−0.005	−1.528, 1.283	.864
Yes_T1_‐No_T2_	0.040	−0.336, 1.500	.214	0.019	−0.650, 1.197	.561
Yes_T1_‐Yes_T2_	0.059	−0.087, 1.270	.087	0.022	−0.472, 0.918	.529
Alcohol drinking						
No_T1_‐No_T2_		Reference			Reference	
No_T1_‐Yes_T2_	0.014	−0.745, 1.173	.662	0.012	−0.763, 1.131	.703
Yes_T1_‐No_T2_	−0.017	−1.082, 0.619	.593	−0.028	−1.219, 0.473	.387
Yes_T1_‐Yes_T2_	0.004	−0.726, 0.809	.916	−0.004	−0.807, 0.729	.920
Exercise						
No_T1_‐No_T2_		Reference			Reference	
No_T1_‐Yes_T2_	0.070	0.072, 1.443	<.05	0.041	−0.247, 1.132	.198
Yes_T1_‐No_T2_	0.030	−0.347, 0.983	.349	0.017	−0.478, 0.834	.593
Yes_T1_‐Yes_T2_	0.042	−0.347, 1.749	.190	0.018	−0.753, 1.355	.558

*Note*: T1 = baseline; T2 = follow‐up; the reference group for lifestyle factors was the No_T1_‐No_T2_ group. Model 1 did not control for any confounding variables; Model 2 controlled for age, sex, education, marital status, economic status, health status, activities of daily living (ADL), instrumental activities of daily living (IADL), abdominal obesity, superoxide dismutase (SOD), total cholesterol (TC), triglyceride (TG) and anemia status at baseline.

### The effects of interactions between lifestyle and anemia on cognitive function change

The effects of interactions between lifestyle factors and anemia on cognitive decline during the follow‐up period were analyzed using multiple linear regressions. Participants with persistent anemia were more likely to experience cognitive decline than nonanemic participants (*β* = −0.174, 95% CI: [−1.746, −0.817], *p <* .001) (Model 1). The effects of interaction between leisure activities and the onset of anemia (No_T1_‐Yes_T2_) on cognitive function changes was significant (*β* = 0.102, 95% CI: [0.188, 0.880], *p <* .01) (Table 3), suggesting that an increased level of participation in leisure activities contributed to delay cognitive decline in older adults who developed anemia during the follow‐up.

After adjusting for confounding variables (Model 2), the results showed that the interactions between leisure activities and anemia on cognitive function changes were still significant (Table 3). Participants with persistent anemia experienced cognitive decline compared with participants who did not have anemia (*β* = −0.118, 95% CI: [−1.362, −0.376], *p <* .01) (Table 3). An increased level of participation in leisure activities delayed cognitive decline in older adults who developed anemia during the follow‐up, compared with those who did not have anemia (*β* = 0.098, 95% CI: [0.174, 0.857], *p <* .01) (Table 3). Additionally, no significant interactions were observed between other lifestyle factors (social activities, exercise, dietary diversity, smoking, and alcohol consumption) and anemia on cognitive function changes (*ps* > .05). The results partly support the second hypothesis that an increased level of participation in leisure activities, but not other lifestyle factors, can buffer the adverse effects of anemia on cognitive function in older adults.

**TABLE 3:** Association of changes in leisure activities and anemia with cognitive function changes.

Variable	Model 1			Model 2		
	*β*	95% CI	*p*	*β*	95% CI	*p*
Anemia						
No_T1_‐No_T2_		Reference			Reference	
No_T1_‐Yes_T2_	−0.018	−1.135, 0.641	.585	−0.002	−0.905, 0.862	.963
Yes_T1_‐No_T2_	−0.026	−0.881, 0.356	.405	−0.010	−0.717, 0.510	.740
Yes_T1_‐Yes_T2_	−0.174	−1.746, −0.817	<.001	−0.118	−1.362, −0.376	<.01
Anemia (No_T1_‐Yes_T2_) * leisure	0.102	0.188, 0.880	<.01	0.098	0.174, 0.857	<.01
Anemia (Yes_T1_‐No_T2_) * leisure	0.015	−0.176, 0.276	.665	0.015	−0.172, 0.273	.659
Anemia (Yes_T1_‐Yes_T2_) * leisure	0.066	−0.020, 0.343	.081	0.060	−0.033, 0.327	.108

*Note*: T1 = baseline; T2 = follow‐up; the reference group for anemia was the No_T1_‐No_T2_ group. Model 1 did not control for confounding variables; Model 2 controlled for age, sex, education, marital status, economic status, health status, activities of daily living (ADL), instrumental activities of daily living (IADL), abdominal obesity, superoxide dismutase (SOD), total cholesterol (TC), and triglyceride (TG) at baseline.

## DISCUSSION

This study investigated the dynamic association between lifestyle changes and cognitive function changes in a nationwide population‐based survey of older Chinese adults. The current results found that increased participation in leisure activities, social activities, and dietary diversity helped delay cognitive decline. While anemia accelerates cognitive decline, older adults who are actively involved in leisure activities can alleviate the negative effects of anemia on cognitive function.

The current study suggests that increased participation in leisure activities, social activities, and dietary diversity in later life can delay cognitive decline. This result is consistent with recent findings by Lee et al. (2020). By scoring lifestyle factors such as smoking, alcohol consumption, exercise, and body weight, these authors found that following a healthy lifestyle or improving an unhealthy lifestyle could help older Korean adults maintain cognitive function or delay cognitive decline (Lee et al., 2020). In the current study, we further explored the effects of changes in specific lifestyle factors on cognitive function, finding that active participation in leisure and social activities and increased dietary diversity could delay cognitive decline for older adults later in life. A high degree of participation in leisure and social activities is beneficial for the maintenance of individuals' cognitive function (Hou et al., 2023; Hou & Li, 2022; Mao et al., 2020; Marioni et al., 2014; Wang, Liang, et al., 2017). While older adults exhibit cognitive decline in a variety of cognitive domains, they experience varying rates and degrees of cognitive decline. Cognitive reserve, a theoretical construct used to explain individual differences in cognitive decline, can explain the above phenomenon (Stern, 2009, 2012). Cognitive reserve may reflect an active process in which the brain actively compensates for neurodegeneration (Stern, 2002). Lifestyle‐enriching activities, including more engagement in social activities, leisure activities, physical activities, technology use, hobbies, and educational activities, are thought to have positive impacts on cognitive function in later life (Hai et al., 2022; Hertzog et al., 2008; Nyberg & Pudas, 2019). Using longitudinal national survey data, the current study reveals dynamic associations between lifestyle changes and cognitive function changes, providing supporting evidence that environmental enrichment could delay cognitive decline in older adults. Moreover, the environmental complexity hypothesis posits that active participation in cognitively and socially intensive activities can provide an environment with rich stimuli and cognitive complexity, which can generate more dendritic branches and synaptic connections in the brain (Greenough et al., 1986; Newson & Kemps, 2005; Schooler & Mulatu, 2001). Leisure and social activities can enrich the environment of older adults, increase synaptic density in the neocortex‐associated cortex, enhance existing synaptic activity, or lead to the formation of more efficient synaptic connection circuits that help these adults compensate for cell loss with cellular gain, increase their cognitive reserve, and enable them to cope better with cognitive decline (Scarmeas & Stern, 2003).

The current results suggest that increasing dietary diversity can help maintain cognitive function in older adults. Recent reviews suggest that following a healthy lifestyle can increase the likelihood of healthy aging by 80%, with healthy eating patterns playing an important role (Dreher, 2018). Because human foods contain multiple nutrients that tend to be interconnected and interact, effective nutritional balance can only be achieved in the context of better dietary diversity; therefore, nutrients that are beneficial for cognitive function, such as folic acid and vitamin C, can work well if dietary diversity is good (Zheng et al., 2021). Low levels of dietary diversity are associated with high levels of oxidative stress, which increases the risk of cognitive impairment (Narmaki et al., 2015; Wang, Zhu, et al., 2017). As older adults age, their ability to resist oxidative stress and maintain normal brain function is greatly reduced (Guidi et al., 2006). A 25‐day intervention study found that those who consumed 600 g of fruit and vegetables daily significantly increased glutathione peroxidase activity and the antioxidant capacity of plasma lipoproteins (Dragsted et al., 2004). Increasing dietary diversity, such as by increasing the intake of fish, fruit, vegetables, and dairy products, can protect the central nervous system and exert certain anti‐inflammatory and antioxidant effects, thereby better protecting cognitive function in older adults (Narmaki et al., 2015).

With increasing age, the ability of digestion and absorption and protein synthesis capacity in older adults decreases, and a low degree of progressive erythropoietic exhaustion may occur; therefore, older adults are prone to anemia (Balducci, 2010). The high incidence of anemia has become an important public health problem. In this study, the proportion of anemia reached 44.4% in older adults. The present study found that persistent anemia exacerbated cognitive decline. Decreased hemoglobin levels lead to a decrease in blood oxygen‐carrying capacity, resulting in continuous hypoxia in the body, which affects the activation of neurons in the brain and leads to cognitive decline (Tan et al., 2018). Animal models have found that erythropoietin receptors are localized in the brain and play a nutritional role in central neurons, and hypoxia caused by anemia reduces erythropoietin levels and increases the risk of neuronal deterioration (Qin et al., 2019). While anemia can impair cognitive function in older adults, a healthy lifestyle can mitigate the negative cognitive effects of anemia in its early stages. For older adults, adherence to a healthy lifestyle, including leisure activities, can help increase cortical thickness and neuron density, reduce the risk of microvascular disease, maintain hippocampal volume and memory capacity, help delay cognitive decline, and contribute to healthy aging (Bennett et al., 2014; Iizuka et al., 2021; Valenzuela et al., 2012).

In contrast to previous studies, our study has not found any effects of changes in smoking and drinking on changes in cognitive function, possibly because the measurement of smoking and drinking is a simple dichotomy that does not take into account changes in specific consumption. While the current study reveals the protective effects of a healthy lifestyle in older adults, it still has some limitations. First, the effects of lifestyle factors on cognitive function and anemia could not be explored over longer follow‐up periods as there were no physiological or anemia data (2017 wave). Second, previous studies have shown that sleep is an important part of lifestyle, and both the length and quality of sleep affect cognitive function in older adults (Ord et al., 2022). However, the database used in this study did not examine this variable. Finally, for a variety of reasons, many participants were not followed in the 2014 wave, which may affect the internal validity of the study to some extent.

In this large‐scale population‐based study, we found that following a healthy lifestyle, such as increased participation in leisure activities, social activities, and dietary diversity, can help older adults alleviate the cognitive decline caused by aging itself and alleviate the adverse effects of anemia on cognitive function. Our findings provide empirical support for public health policy and individualized recommendations for older adults seeking to maintain cognitive function. Future studies are warranted to further explore the effects of combined interventions targeting multiple lifestyle factors on cognitive function in order to elucidate the role of lifestyle in achieving healthy aging.

## FUNDING INFORMATION

This work was supported by the National Natural Science Foundation of China (31871143). The funders played no role in the design, conduct, or reporting of this study.

## CONFLICT OF INTEREST STATEMENT

The authors declare that they have no conflicts of interest with respect to the research, authorship, and/or publication of this paper.

## ETHICS STATEMENT

The CLHLS obtained ethics approval from the Research Ethics Committee of Peking University (IRB00001052‐13074) and received written informed consent from all participants.

## Data Availability

Data were obtained from the Chinese Longitudinal Healthy Longevity Survey (CLHLS) (https://opendata.pku.edu.cn/dataverse/CHADS).
